# Barriers, Benefits, and Beliefs of Brain Training Smartphone Apps: An Internet Survey of Younger US Consumers

**DOI:** 10.3389/fnhum.2016.00180

**Published:** 2016-04-20

**Authors:** John Torous, Patrick Staples, Elizabeth Fenstermacher, Jason Dean, Matcheri Keshavan

**Affiliations:** ^1^Department of Psychiatry, Beth Israel Deaconess Medical CenterBoston, MA, USA; ^2^Department of Biostatistics, Harvard T.H. Chan School of Public HealthBoston, MA, USA

**Keywords:** brain, apps, smartphones, memory, technology assessment

## Abstract

**Background**: While clinical evidence for the efficacy of brain training remains in question, numerous smartphone applications (apps) already offer brain training directly to consumers. Little is known about why consumers choose to download these apps, how they use them, and what benefits they perceive. Given the high rates of smartphone ownership in those with internet access and the younger demographics, we chose to approach this question first with a general population survey that would capture primarily this demographic.

**Method**: We conducted an online internet-based survey of the US population via mTurk regarding their use, experience, and perceptions of brain training apps. There were no exclusion criteria to partake although internet access was required. Respondents were paid 20 cents for completing each survey. The survey was offered for a 2-week period in September 2015.

**Results**: 3125 individuals completed the survey and over half of these were under age 30. Responses did not significantly vary by gender. The brain training app most frequently used was Lumosity. Belief that a brain-training app could help with thinking was strongly correlated with belief it could also help with attention, memory, and even mood. Beliefs of those who had never used brain-training apps were similar to those who had used them. Respondents felt that data security and lack of endorsement from a clinician were the two least important barriers to use.

**Discussion**: Results suggest a high level of interest in brain training apps among the US public, especially those in younger demographics. The stability of positive perception of these apps among app-naïve and app-exposed participants suggests an important role of user expectations in influencing use and experience of these apps. The low concern about data security and lack of clinician endorsement suggest apps are not being utilized in clinical settings. However, the public’s interest in the effectiveness of apps suggests a common theme with the scientific community’s concerns about direct to consumer brain training programs.

## Introduction

Over the last decade, consumer markets have seen a veritable explosion in products marketed for “brain training”. “Brain training’ entails the use of specific exercises, often games, which reputedly improve cognitive performance. While many companies advertise a neuroscientific basis for the efficacy of their brain training products, there is little peer-reviewed research to substantiate these claims. Nevertheless, brain training exercises have maintained broad appeal. Current estimates suggest that brain training has become a billion dollar annual industry, with over 70 million active users of Lumosity, one of the most popular brain training programs. (Sukel, 2015). A direct evaluation of these programs” efficacy is beyond the scope of this article, and we refer the reader to other work which has addressed this topic (Owen et al., 2010; Bavelier et al., 2012; Rabipour and Raz, 2012; A Consensus on the Brain Training Industry from the Scientific Community, 2014; Lampit et al., 2014; Toril et al., 2014; Ballesteros et al., 2015). This article seeks to assess consumer motivations and perceived benefits and attitudes towards of brain training exercise programs, with a particular focus on smartphone applications (apps). Smartphone apps offer an accessible, affordable, and convenient method for millions of consumers to access and engage in brain training. Understanding why consumers choose to download and use brain training apps is an important question that can help clinicians discuss and understand the role of these digital tools. As non-invasive, easily accessible, and affordable cognitive interventions, brain training apps are appealing consumer devices. From younger workers hoping to become more efficient (Borness et al., 2013), to older adults concerned about dementia (Corbett et al., 2015; Salthouse, 2015) and other psychiatric disorders (Keshavan et al., 2014), brain training holds tremendous promise.

There is little research or consensus regarding what drives consumers to use brain-training apps. One recent study investigated user expectations and noted that people who use these apps tend to have high expectations, even before using them (Rabipour and Davidson, 2015). In early 2016, the US Federal Trade Commission ordered one brain training app, Lumosity, to pay a two million dollar settlement regarding “deceptive advertising” stating the company “preyed on consumers” (Lumosity to Pay $2 Million to Settle FTC Deceptive Advertising Charges for Its “Brain Training” Program [Internet]., 2016). However, little is known about who uses brain training apps, which apps they are using, what they expect in terms of cognitive improvement, and what they perceive as barriers to use.

Although brain training apps are marketed across all ages, we chose to focus on a more tech-savvy demographic, which was highly correlated with a younger demographic, which has also been shown to be the largest group* per capita* of smartphone owners. National survey data suggests that 85% of US adults between ages 18–29 and 70% between ages 30–50 own a smartphone (Smith, 2015). Compared to those over the age of 50, this younger demographic is more than twice as likely to use their phones to find health related information online (Fox and Duggan, 2012). Survey data of outpatient psychiatric patients also found high rates of smartphone ownership and interest in apps among a similar demographic (Torous et al., 2014), though there is no survey data on smartphone ownership and app interest among those seeking brain training. We conducted the following survey study in order to better characterize consumer opinions and attitudes towards brain training apps specifically choosing a survey modality that would capture the largest tech-savvy group of consumers through an on-line survey format as this demographic is most likely to use these programs. This tech-savvy group of consumers is largely comprised of a younger demographic and the majority of our respondents were under the age of 50.

## Materials and Methods

**Figure 1:**
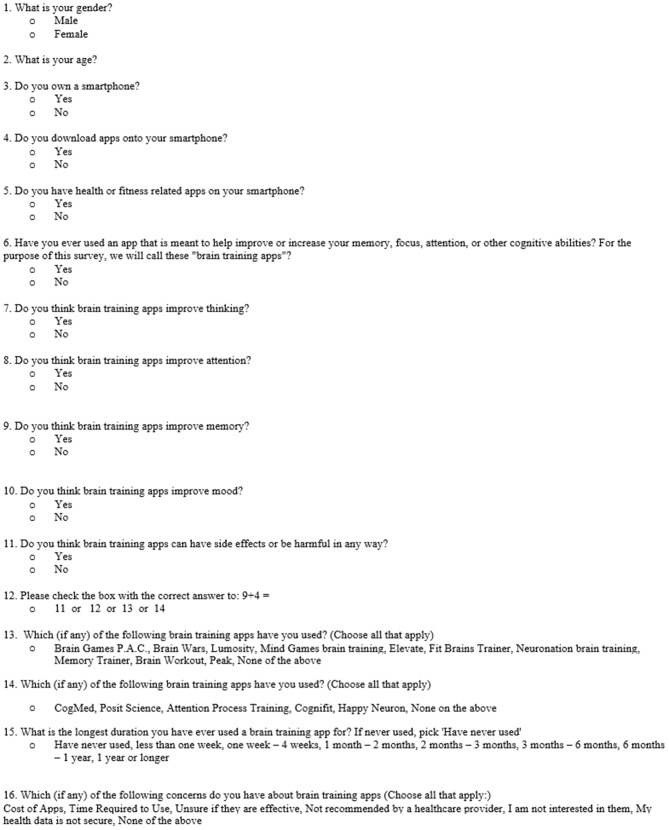
**A copy of the survey questions reformatted to be displayed in a single figure**.

In order to reach a large population, we conducted a survey study of the general United States population, using an online survey platform, Amazon’s Mechanical Turk (mTurk). This platform has been validated for more complex behavioral research (Crump et al., 2013), though here we used it as a simple anonymous survey platform. The survey was conducted in September 2015 and was offered to 3125 subjects registered to take surveys on mTurk, with compensation of 20 cents per survey. The survey, shown below in Figure 1, received hospital IRB approval. Of note, we included a simple math question, “9 + 4 = ?” in order to ensure that subjects were actively engaged with the material and not simply “clicking” through the survey.

As our survey contained questions around which brain training apps subjects had used, we sought to identify the most popular brain training apps from both the Apple iTunes store and Android Google Play store. Considering that each app store ranks apps by different criteria and provides different information on number of downloads, reviews, and users, we combined the top ten apps from each marketplace (in June 2015) in a single top ten list, based on our judgment and consensus. We also sought to identify brain-training apps that have been clinically studied and identified six apps from an article that reviewed the evidence for brain training apps (Brooks, 2014). Lumosity was the only app the overlapped as it has been clinically studied and was in the top ten apps on the commercial marketplaces. Some questions (7, 8, 9, 10, 11) asked subjects for their perception on features of brain training regardless of use of these apps and this was intentional to be able to explore how perception varied with use. In creating the survey, we composed a list of possible concerns of the participants in the survey (see Question 16). We interpreted these concerns as possible barriers. While our survey was open to anyone and age was not an exclusion factor, the mTurk platform is skewed towards a more tech savvy and therefore younger demographic.

## Results

**Figure 2:**
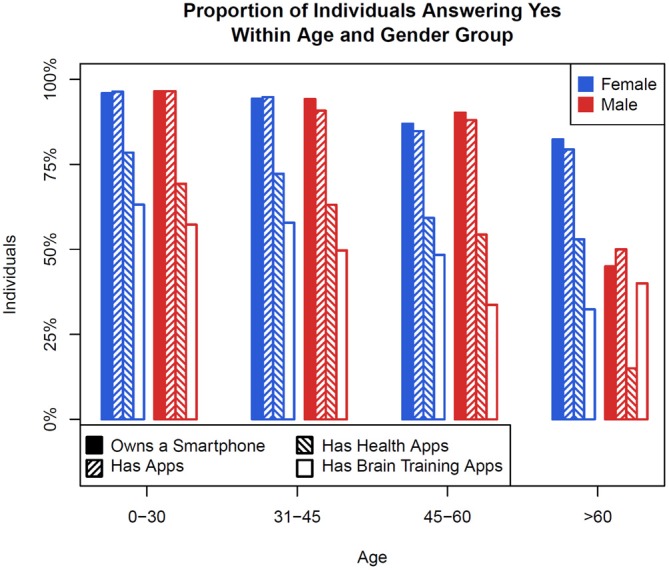
**A bar graph showing the proportion of survey respondents by age and sex who own a smartphone, have apps, have healthcare apps, and have brain training apps**.

Over a 2-week period in September 2015, 3125 subjects completed the survey. 48.4% were female (age mean 33.9, SD 12.2), 51.3% were male (age mean 30.9, SD 9.2), and 0.3% of respondents did not answer this question. Of the 3125 subjects, 1558 (nearly 50%) were under age 30, 978 (just over 31%) were between ages 31 – 45, 276 (almost 9%) between ages 46 – 60, and 54 (nearly 2%) older than age 60. Figure 2 below shows the a breakdown of smartphone ownership, having any apps, having health apps, and having brain training apps by age brackets. 93.7% of subjects report having apps, 69.2% having used health apps, and 55.7% having used brain training apps. 66.9% reported that brain training apps helped with thinking, 69.3% with attention, 53.3% with mood, 65% with memory, and 14.9% reported that they felt there may be dangers with app use. 98.2% answered the math question correctly. Demographic age and gender related information of subjects and there ownership/use of smartphones and apps is shown below in Figure 2.

**Figure 3:**
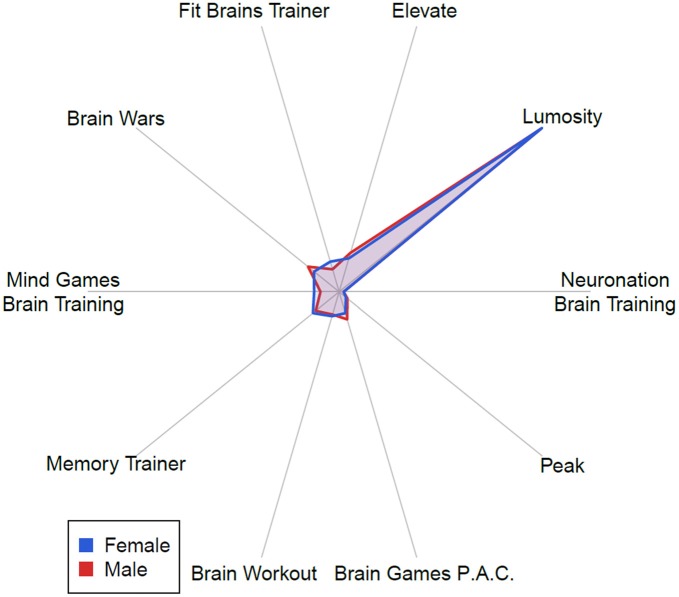
**A polar plot showing the proportion of brain training apps used, stratified by reported gender.** Each gender is normalized to show the same total volume, and the magnitude is normalized to show the global maximum of reported proportion at the maximum radius. Lumosity is clearly reported as used more than any other brain training app.

Of the 16 apps subjects were asked if they had ever used, Lumosity was the most used with 70% of those who had used brain training apps having tried it. Figure 3 below displays apps used by subjects in a polar plot showing the proportion of brain training apps used, stratified by reported gender.

Looking at Figure 3, we notice several trends. First, the largest correlations (0.77 – 0.82) exist between beliefs about the positive effects of brain training apps on cognitive abilities (memory, thinking, and attention). A strong but attenuated correlation also exists between these and the reported benefit of brain training apps on mood (0.34 – 0.40). However, it is instructive to observe that the correlation between using brain apps and reporting the positive effects above, while statistically significant, is very slight (0.09 – 0.14). This suggests two points: first, that those who use brain training apps do not observe the main benefits intended by the app; and second, those who do report benefits report many that are mostly independent of each other, and do not distinguish strongly between specific benefits.

**Figure 4:**
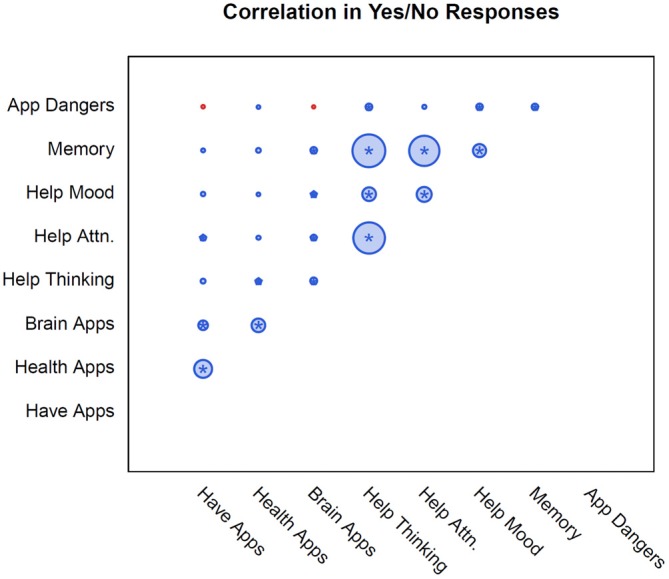
**The pairwise correlations between each response.** Correlation is proportional to area and colored by valence (positive shown in blue, negative in red), with the maximum correlation being 0.82 (Memory and Help Thinking). Statistical significance (adjusted for multiple testing) is indicated with a star.

In order to better understand correlations between individual survey responses, we calculated the correlation coefficients which are displayed below in Figure 4.

**Figure 5:**
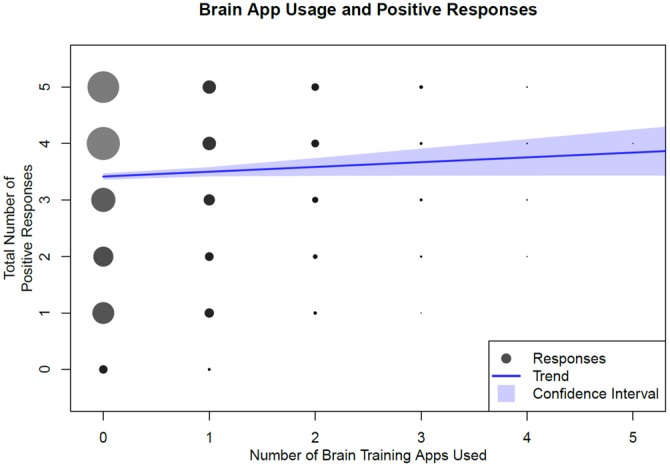
**Total number of brain training apps used by total number of responses.** We consider a positive response to be an answer “Yes” to Questions 7–10, and “No” to Question 11. The blue line is a linear regression fit, with 95% confidence bands.

**Figure 6:**
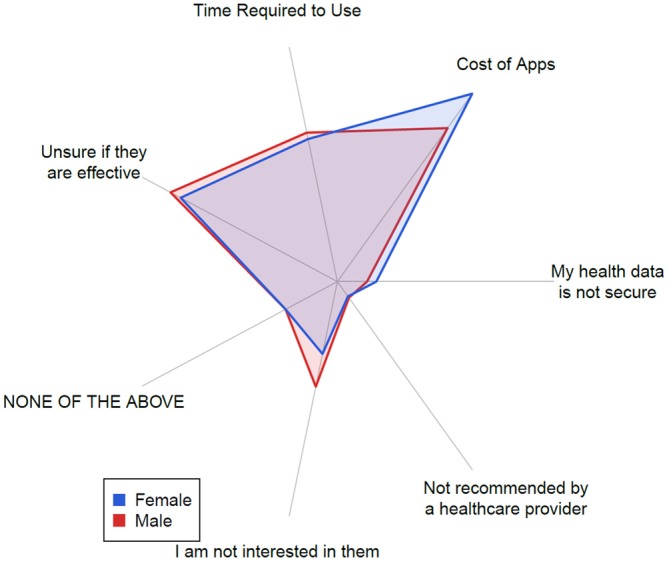
**A polar plot showing the proportion of barriers to use reported, stratified by reported gender.** Each gender is normalized to show the same total volume, and the magnitude is normalized to show the global maximum of reported proportion at the maximum radius.

To understand the summary difference between those who have not used app vs. those who have used one or more, we created a score calculated by adding one each point for responding “yes” to any of Questions 7–10 (apps help with either attention, memory, thinking, or mood) and for responding “no” to Question 11 (there are dangers to app use). Thus the potential score range is between 0 and 5. The results are displayed below in Figure 5 which presents perceptions of brain training apps in comparison to the number of brain training apps a subject has reported using, subjects were asked to report barriers toward brain training app use and results are shown below in Figure 6.

Figure 6 shows that our sample is not generally concerned about the security of the data gathered by a brain training app (4% males, 6% females), nor whether the brain training app was recommended by a healthcare provider (3% females, 2% males). In contrast, subjects were most likely to report concerns about the cost of apps (30% females, 25% males), as well as concerns about their effectiveness (25% females, 26% males). To understand the association between app ownership on perceptions of efficacy, we compared the distribution of the number of brain training apps used among those who responded that they have concerns about app efficacy to those who did not respond that they have these concerns. A *t*-test for a difference in the means of these distributions yielded a p-value of 0.12, failing to reject the hypothesis that those with or without concerns about app efficacy differ in the mean number of brain-training apps they use. We also conducted a similar *t*-test to understand if there is an association of app ownership with concerns about cost of apps, and again failed to find a significant difference (*p* = 0.35).

## Discussion

To date, this is the largest Internet survey of user perceptions of brain training apps, which provides a window into the use, barriers, and consumer attitudes towards these programs. The mean age of the 3125 respondents was 32.4 years old, which is consistent with the largest demographic of smartphone owners, app users, and internet survey participants. Our data elucidates strong positive perceptions of cognitive training apps, with roughly equal percentages of respondents reporting a belief that these apps improve thinking (66.9%), memory (70.3%), and attention (69.3%). A significant proportion (53.3%) of respondents also believed that brain training apps have a positive effect on mood.

While nearly 50% of survey respondents were under age 30, our results still provide an interesting window on who is using brain training apps. Rates of smartphone ownership, having health apps, and having brain training apps was very similar although slightly lower in the 31 – 45 age demographic as compared to those less than 30, suggesting a broader appeal of these apps beyond those less than age 30. Given that only 9% of respondents were in the 45 – 60 demographic and 2% in the above 60 years old demographic it is harder to interpret the results for these groups. This 2% result is interesting as it is in line with a recent survey study suggesting that only 1.2% of US adults over age 65 have ever used a handheld device to track their health (Shahrokni et al., 2015). Given national trends that smartphone ownership in younger generations is reaching saturation, and our results that ownership is also less in older demographics, it is possible that the next wave of growth in brain training apps may come from those who are older as they begin to further adopt smartphones Although our sample size is small for adults over age 60, it is interesting to note that in male subjects, brain training apps were reported downloaded more than health apps. While our study is not designed to answer why this may be so, it suggests that brain training apps may be of strong interest in this population.

Subjects’ belief that these apps are beneficial for thinking is strongly correlated with the belief that they improve attention, memory and mood. However, this correlation was not as strong in users who reported prior use of a brain training app. This finding is in line with perceptions of barriers in that using a greater number of apps did not improve consumer attitudes. Those who had never used a brain training app reported a positive response score of 3.5. There was a small positive correlation between the number of apps used and positive responses, with the positive response score increasing only minimally for those who had used more apps, reaching 3.84 for those who had used up to five apps. Regardless of the number of apps used, the use of these programs was not strongly correlated with a change in the positive response score. These results are thus in agreement with a recent study, which found that consumers have high expectations for brain training apps before their use (Rabipour and Davidson, 2015). In addition, we have shown that consumer expectations and perceptions do not change after these apps have been used. It is possible that the positive attitude towards these programs may stem primarily from preexisting expectations, not positive experiences with the apps themselves. Or conversely, that strong preexisting positive expectations may be the primary driving force behind app ownership and usage. Our results also suggest that consumer opinions on barriers to app use, such as cost and perceived efficacy, are not statistically correlated with app ownership or number of apps used by subjects. These seemingly paradoxical results may reflect consumers’ inconsistency in the recognition of their own preferences, and highlight the complexity in understanding why consumers choose to use or not use these apps.

Whereas clinicians are concerned with privacy and clinical recommendations and evidence backing new technologies like apps (Huckvale et al., 2015) these features were of least concern to consumers, who cited cost as the primary barrier to use. Of note, consumers also listed uncertainty regarding the efficacy of these programs as a strong barrier to use. Overall, our results suggest that consumers prefer an app that is inexpensive, time efficient, and has an evidence base to support its efficacy. Although our survey did not specify what was meant by health data not being secure, further research could explore the overall lack of concern regarding data security. For instance, it is possible that consumers are unaware of how their healthcare data may be used when using these apps.

While our survey did not assess whether user perceptions of efficacy correlates with actual benefit, this topic has recently become a topic of debate (Brooks, 2014; Rabipour and Davidson, 2015). A recent consensus statement by numerous neuroscientists further underscores the lack of rigorous scientific evidence and the concern for misleading marketing (A Consensus on the Brain Training Industry from the Scientific Community, 2014). While many studies have shown positive results (Green and Bavelier, 2008; Anguera et al., 2013; Ballesteros et al., 2014; Hardy et al., 2015), there is concern that these improvements could also be related to improved skill at using the app, rather than an actual improvement in cognition (Owen et al., 2010; Burch, 2014). Our results, which suggest that positive consumer attitudes are related more to preexisting beliefs than to positive user experiences, could support the notion of a digital placebo effect. Our results also indicate a high demand for a more rigorous, scientific approach to these applications. Continued consumer demand for these applications, despite the current paucity of evidence, could present an opportunity for academic researchers, consumers, and industry to collaborate in an exploration of new approaches to brain training.

Like all survey research, our study has several limitations. Our results are self-reported and many questions, especially around barriers were subjective. We focused on correlations, and while we can speculate on potential links between these correlations, our survey was not designed to address causation. In addition, our data is inevitably skewed towards a younger and more digitally connected population, as our research was conducted online and targeted US residents. This limitation was expected, given our focus on tech-savvy internet users and the use of the mTurk platform. Our survey was not designed to assess if they were seeking brain training apps for a specific reason, e.g., ADHD symptoms. Although our respondents reported high rates of smartphone ownership, 96% for those below age 30, such numbers are close to the US national average which in 2015 was reported at 85% for this same demographic. While our survey may over represent younger connected individuals, it underrepresents older adults (age >65) as they only represented 54 of our 3125 subjects or 1.7%. Perceptions of brain training in an older demographic is an important future research direction, as many older adults may use brain training apps to address declining cognition. Another potential limitation of survey data in general is poor attention to the online survey task, but the fact that over 98% of respondents answered the distracter math question correctly suggests that they were attentive to the other survey questions as well. Of note, we included response from the slightly less than 2% of respondents who answered the math question incorrectly.

The future of brain training smartphone apps is at a crossroads. One path leads to further development of brain training apps, driven largely by marketing and expectations, rather than scientific evidence. The other route rests on further app development, with a focus on efficacy research and generalizable benefits. Based on our survey data of consumer perspectives and the current body of scientific literature, it appears that brain training app users would prefer the latter path. With the right scientific efforts, consumer education and empowerment, and partnerships with industry, this goal will hopefully be attainable in the near future.

## Author Contributions

JT and MK conceived the research idea. JT, EF, and JD wrote the protocol and IRB. PS analyzed the data and produced all figures. JT, EF, JD, and MK conducted background literature review. All authors helped in the writing and drafting on this manuscript. All authors edited the manuscript.

## Funding

PS is supported by NIH Grant 2T32AI007358-26 (PI Pagano).

## Conflict of Interest Statement

The authors declare that the research was conducted in the absence of any commercial or financial relationships that could be construed as a potential conflict of interest. The reviewer JMR and handling Editor SB declared their shared affiliation, and the handling Editor states that the process nevertheless met the standards of a fair and objective review.
