# Corrigendum: Barriers, Benefits, and Beliefs of Brain Training Smartphone Apps: An Internet Survey of Younger US Consumers

**DOI:** 10.3389/fnhum.2016.00253

**Published:** 2016-06-02

**Authors:** John Torous, Patrick Staples, Elizabeth Fenstermacher, Jason Dean, Matcheri Keshavan

**Affiliations:** ^1^Department of Psychiatry, Beth Israel Deaconess Medical CenterBoston, MA, USA; ^2^Department of Biostatistics, Harvard T.H. Chan School of Public HealthBoston, MA, USA

**Keywords:** brain, apps, smartphones, memory, technology assessment

**Reason for Corrigendum**: Addition of conflict of interest statement by Dr. Matcheri Keshavam.

**Clearly state the mistake being fixed**.

After publication, Dr. Matcheri Keshavan noted the paper should include this statement “MK has a contract to purchase Lumosity services for one of his studies, and has provided consultant services to Forum Pharmaceuticals.”

## Author contributions

JT and MK conceived the research idea. JT, EF, and JD wrote the protocol and IRB. PS analyzed the data and produced all figures. JT, EF, JD, and MK conducted background literature review. All authors helped in the writing and drafting on this manuscript. All authors edited the manuscript.

### Conflict of interest statement

The authors declare that the research was conducted in the absence of any commercial or financial relationships that could be construed as a potential conflict of interest. MK has a contract to purchase Lumosity services for one of his studies, and has provided consultant services to Forum Pharmaceuticals.

